# Deoxyglucose prevents neurodegeneration in culture by eliminating microglia

**DOI:** 10.1186/1742-2094-11-58

**Published:** 2014-03-26

**Authors:** Anna Vilalta, Guy C Brown

**Affiliations:** 1 Department of Biochemistry, University of Cambridge, Tennis Court Road, Cambridge CB2 1QW, UK

**Keywords:** Neurodegeneration, Glycolysis, Neuroinflammation, Brain trauma, Brain ischemia, Alzheimer’s disease, Energetics, Glia, Microglia, Glucose

## Abstract

**Background:**

2-Deoxy-d-glucose is an inhibitor of glycolysis, which is protective in animal models of brain pathology, but the mechanisms of this protection are unclear. We examined whether, when and how deoxyglucose protects neurons in co-culture with astrocytes and microglia. Microglia are brain macrophages, which can damage neurons in inflammatory conditions.

**Methods:**

Deoxyglucose was added to primary cultures of microglia and astrocytes from rat cortex, or neurons and glia from rat cerebellum, or the BV-2 microglial cell line, and cell death and cell functions were evaluated.

**Results:**

Surprisingly, addition of deoxyglucose induced microglial loss and prevented spontaneous neuronal loss in long-term cultures of neurons and glia, while elimination of microglia by l-leucine-methyl ester prevented the deoxyglucose-induced neuroprotection. Deoxyglucose also prevented neuronal loss induced by addition of amyloid beta or disrupted neurons (culture models of Alzheimer’s disease and brain trauma respectively). However, deoxyglucose greatly increased the neuronal death induced by hypoxia. Addition of deoxyglucose to pure microglia induced necrosis and loss, preceded by rapid ATP depletion and followed by phagocytosis of the microglia. Deoxyglucose did not kill astrocytes or neurons.

**Conclusions:**

We conclude that deoxyglucose causes microglial loss by ATP depletion, and this can protect neurons from neurodegeneration, except in conditions of hypoxia. Deoxyglucose may thus be beneficial in brain pathologies mediated by microglia, including brain trauma, but not where hypoxia is involved.

## Background

2-Deoxy-d-glucose (DOG) is a glucose analogue that enters cells and the brain via glucose transporters, and is phosphorylated by hexokinase to deoxyglucose-6-phosphate, which then inhibits glycolysis at hexokinase and glucose-6-phosphate isomerase [1]. DOG can thus kill cells that are reliant on glycolysis, such as some cancer cells, via ATP depletion without killing cells that can derive sufficient energy from mitochondria, such as neurons [2]. DOG has been extensively tested as a potential therapeutic agent in cancer specifically killing tumors cells via inhibition of glycolysis [1,2].

Perhaps more surprisingly, DOG can prevent neurodegeneration in the brains of animals subjected to a variety of insults, including chemical or electrical induction of epileptic seizures [3,4], a neurotoxin (iminodipropionitrile) [5], brain radiotherapy [6], an inducer of Parkinsonism (1-methyl-4-phenyl-1,2,3,6-tetrahydropyridine) [7] and a triple transgenic model of Alzheimer’s disease [8]. DOG rapidly enters the brain and brain cells of healthy humans, and thus its derivatives are widely used to image brain metabolism [9]. DOG is also reasonably well tolerated by the body at concentrations that protect the brain [6,10]. DOG has thus been proposed as a therapy for a broad range of brain pathologies and even ageing [11], but the mechanism by which DOG protects the brain is unclear.

We investigate here whether DOG is protective or toxic to neurons in culture, and the mechanisms involved. Surprisingly, we find that DOG is very protective to neurons in a variety of conditions by depleting microglia, but, unsurprisingly, is toxic to neurons in hypoxic conditions.

Microglial cells are specialized macrophages of the central nervous system [12]. Once activated by inflammatory or pathological changes in the surrounding microenvironment microglia can become toxic and/or protective to nearby neurons [13-15]. The neurotoxicity of activated microglia may contribute to a variety of brain pathologies such as stroke, trauma, meningitis, epilepsy, motor neuron disease, Parkinson’s disease and Alzheimer’s disease [13,15].

Alzheimer’s disease is characterized by amyloid plaques (mainly consisting of aggregated amyloid beta (Aβ)) and tau tangles, accompanied by microglial activation and progressive loss of neurons and synapses, which are thought to cause the progressive dementia. Micromolar levels of Aβ can induce direct neurotoxicity, but nanomolar levels of Aβ induce neuronal loss mediated by microglia, so that removal of microglia prevents Aβ-induced neurotoxicity [16,17]. Brain trauma can also invoke secondary, delayed neuronal damage mediated by microglia [18,19]. In brain slices or long-term cultures of microglia and neurons, spontaneous loss of neurons mediated by microglia can also occur if microglial activation is not restrained [20,21]. Understanding how to limit microglial neurotoxicity may thus be important in treating a wide variety of brain pathologies.

The cellular bioenergetics of microglia has been little studied, and this is the first report that inhibition of glycolysis with DOG can kill microglia.

## Methods

### Materials

All chemicals and reagents were purchased from Sigma (St Louis, MO, USA) except cell culture reagents (PAA, Piscataway, NJ, USA), Dulbecco’s modified Eagle’s medium (Invitrogen, Carlsbad, CA, USA), Aβ_1–42_ (EZBiolab, Carmel, IN, USA), Alexa fluor-488-labelled Griffonia Simplicifolia isolectin B_4_ (Invitrogen), BCA protein assay (Pierce, Rockford, IL, USA), rat tumor necrosis factor alpha (TNFα) quantikine enzyme-linked immunosorbent assay kit (R&D Systems, Minneapolis, MN, USA), ATP Bioluminescence Assay Kit (Roche, Basel, Switzerland), ZVal-Ala-d,l-Asp(OMe)-fluoromethylketone and bocaspartyl (OMe)-fluoromethylketone (Bachem, Bubendorf, Switzerland), and 5 μm fluorescent microspheres (Spherotec, Lake Forest, IL, USA).

### Cell culture

All experiments were performed in accordance with the UK Animals (Scientific Procedures) Act (1986) and were approved by the Cambridge University local ethical committee. Primary mixed neuronal/glial cultures were prepared from cerebella of postnatal day 5 to 7 Wistar rats (male and female) as described previously [22]. The approximate composition of the mixed cultures is 85 ± 5% neurons, 7 ± 3% astrocytes, and 5 ± 3% microglia. Neurons were plated at a density of 5 × 10^5^ cells/24-well plate. Pure microglia and glial cultures were prepared as described previously [23]. Glial cells were plated at a density of 2 × 10^5^ cells/24-well plate. Glial cells were also seeded at a high density into 75 cm^3^ flasks to allow the extraction of pure microglia at 7 to 9 days *in vitro* (DIV). Microglia-depleted cultures were obtained by adding 50 mM l-leucine-methyl ester (LME) for 4 hours [24,25]. The murine microglial cell line BV-2 (passage < 30) was maintained in a glia medium consisting of Dulbecco’s modified Eagle’s medium (Invitrogen) supplemented with 10% fetal bovine serum (PAA) in 75 cm^2^ flasks (Nalge Nunc, Penfield, New York, USA) at 37°C, 5% carbon dioxide.

### Cell treatments

Cultures were treated at 7 to 9 DIV. Cells were treated with 250 nM Aβ_1–42_ prepared as described previously [17]. DOG was used at a final concentration of 10 mM. Cytochalasin D was added at a final concentration of 1 μM for 6 hours.

### Disrupted neurons

Addition of disrupted neurons to mixed neuronal–glial cultures was used to model brain trauma. Disrupted neurons were obtained by taking a live neuronal–glial culture (prepared as above, mostly neurons), replacing the culture medium with a low volume of phosphate-buffered saline, scratching the cells with a cell scraper to disrupt processes, scraping the cells to displace them from the surface, and passing cells 10 times through a 0.4 mm × 13 mm syringe needle. The total protein content was measured by BCA protein assay (Pierce). Then 30 μg disrupted neurons (approximately 1.7 × 10^5^ cells) were added to each well of live neuronal–glial cultures in a 24-well plate to mimic traumatic brain injury.

### Hypoxia treatment

Neuronal–glial cultures were placed in a Modular Incubator Chamber (MIC-101; Billups-Rothenberg, San Diego, CA, USA) and the chamber was flushed either with a hypoxic gas mixture (2% oxygen, 5% carbon dioxide, 93% nitrogen) and sealed or left in normoxic conditions (21% oxygen, 5% carbon dioxide, 74% nitrogen), and both of these samples placed in the same carbon dioxide incubator at 37°C for 12 hours. The percentage of oxygen was checked with an oxymeter at the beginning and the end of the hypoxia period. At the end of the hypoxic or normoxic period, plates from the hypoxic chamber were removed and placed in the incubator for reoxygenation for 4 days together with normoxic plates.

### Phagocytosis assay

Pure microglial cultures were plated at a density of 1 × 10^5^ cells in poly-l-lysine-coated 24-well plates and treated with 10 mM DOG for 21 hours at 37°C. After 21 hours, 5 μm carboxylate-modified latex microspheres were added (diluted 1:2) for 2 hours and then washed with phosphate-buffered saline to remove the excess. Images were taken with a Leica DMI 6000 microscope (Leica, Solms, Germany) and bead uptake was evaluated.

### Cell density quantification

Cell densities (number of live neurons, live microglia, apoptotic and necrotic cells) were assessed in cultures at different time points depending on the experiment. For live cell counts, cultures were incubated for 20 minutes with the nuclear stains Hoechst 33342 (10 μg/ml, to stain nuclei), propidium iodide (1 μg/ml, to stain nuclei of necrotic cells) and Alexa 488-tagged isolectin-B4 (1 μg/ml, to stain microglia). Cell densities were assessed using a Leica DMI 6000 microscope (20× magnification, field size = 1.9 × 10^5^ μm^2^; Leica). Apoptotic neurons were identified by chromatin condensation of their nuclei. Necrotic cells were identified by propidium iodide staining of their nuclei. Only propidium iodide-negative cells with nuclear condensation were counted as apoptotic. Cells that were not necrotic or apoptotic were counted as live. Microglia were identified by isolectin-B4 staining. Astrocytes were identified by flat shape and large, bean-shaped nuclear morphology. Neurons were identified by small, round cell bodies and neuronal processes.

### TNFα measurement

The inflammatory mediator TNFα was measured using a rat TNFα quantikine enzyme-linked immunosorbent assay kit (R&D Systems) according to the provided protocol. Values were expressed as picograms per milliliter.

### ATP measurement

ATP was determined luminometrically (Jade luminometer; Labtech International, Uckfield, East Sussex, UK) using an ATP Bioluminescence Assay Kit (Roche) according to the provided protocol. Briefly, pure microglia was used 24 hours after plating, at a density of 1 × 10^5^ cells/well in poly-l-lysine-coated 24-well plates. Before treatment, culture medium was removed to leave 200 μl/well and then 10 mM DOG was added for 1 hour. The same volume of lysis buffer was then added, and 100 μl of the mix added to 100 μl luciferase in the luminometer and the light emission measured. ATP concentrations were expressed as relative light units.

### Flow cytometry

Viability of BV-2 cells exposed to 10 mM DOG for 0, 3, 6, 24 or 48 hours was analyzed by flow cytometry (BD Accuri C6 flow cytometer; BD, Franklin Lakes, NJ, USA) with Annexin V (fluorescein isothiocyanate labeled) and propidium iodide. Then 10,000 events were collected per well and two wells per condition in at least three independent experiments to quantify the total number of cells, the number of Annexin V-positive cells (both propidium iodide-positive and propidium iodide-negative) and the number of propidium iodide-positive cells (both Annexin V-positive and Annexin V-negative).

### Statistics

Four microscopic fields per well in two wells per condition were quantified in at least three independent experiments (on different cultures from different animals). Statistical analysis was performed using SPSS software (SPSS Inc., Chicago, IL, USA). Normality of data was verified by Kolmogorov–Smirnov test. Means among groups were compared by one-way analysis of variance and *post-hoc* Bonferroni test when data were normally distributed. If the data were not normally distributed, then the Kruskal–Wallis test was used to compare differences among groups. The Mann–Whitney *U* test was used to determine significant differences between pairs of variables that did not follow a normal distribution, and the *t* test for normally distributed data. Graphs are expressed as means ± standard error of the mean. Results were considered significant at *P* < 0.05.

## Results

### Deoxyglucose prevents neuronal loss mediated by microglia

**Figure 1:**
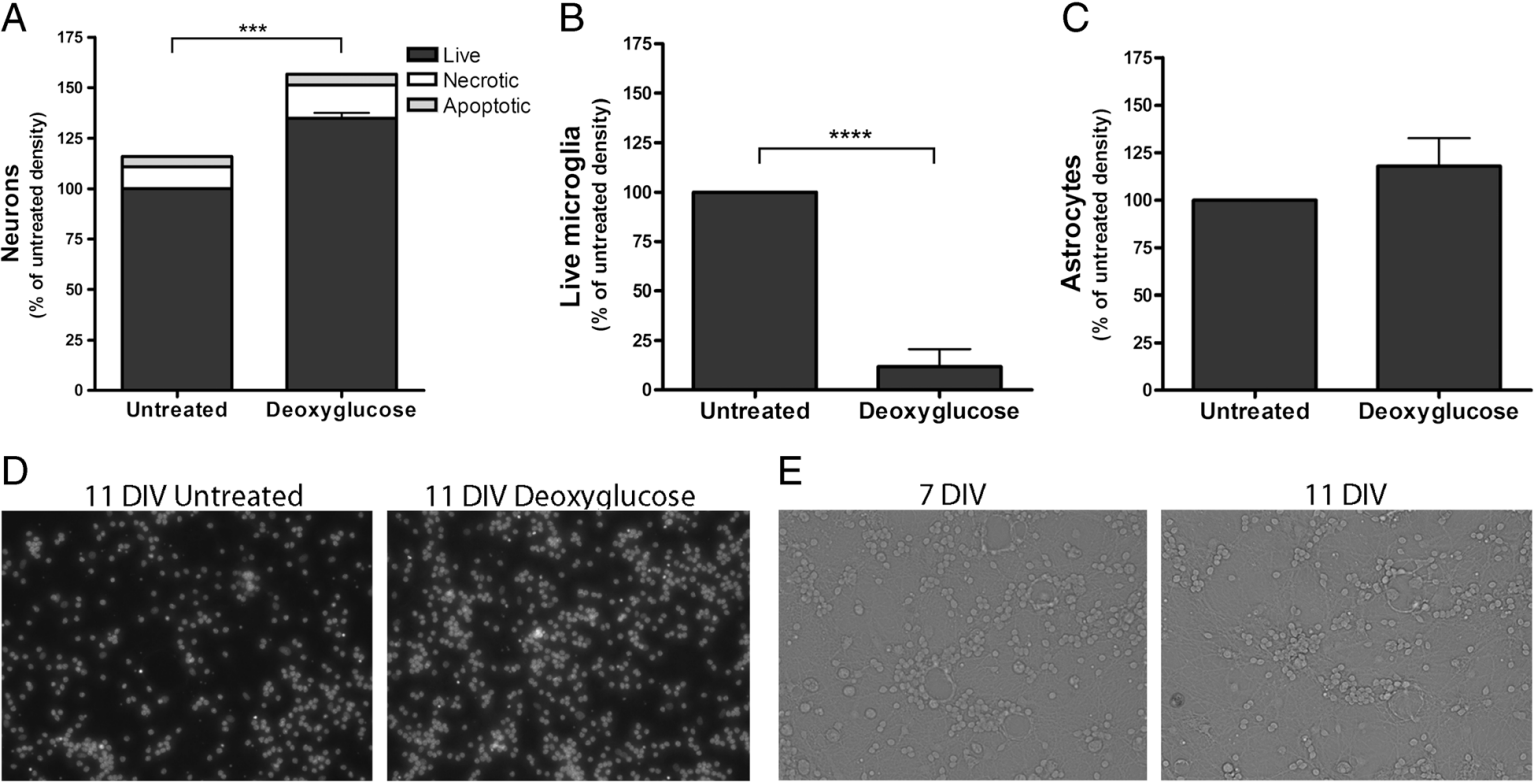
**Deoxyglucose prevents spontaneous neuronal loss *in vitro. *(A)** Density of live, necrotic and apoptotic neurons in neuronal–glial cultures at 11 days *in vitro* (DIV) depicted ±10 mM deoxyglucose added at 7 DIV. **(B)** Density of live microglia in the same experiment. **(C)** Density of live astrocyes in the same experiment. **(D)** Sample fluorescent images of the same experiment stained with Hoescht 33342 to identify nuclei. **(E)** The same untreated field imaged in phase contrast brightfield at 7 and 11 DIV, showing neuronal loss. Data presented as mean ± standard error of the mean for ≥ 3 independent experiments. Statistically significant differences between live neuronal densities: ***P < 0.001, ****P < 0.0001.

**Figure 2:**
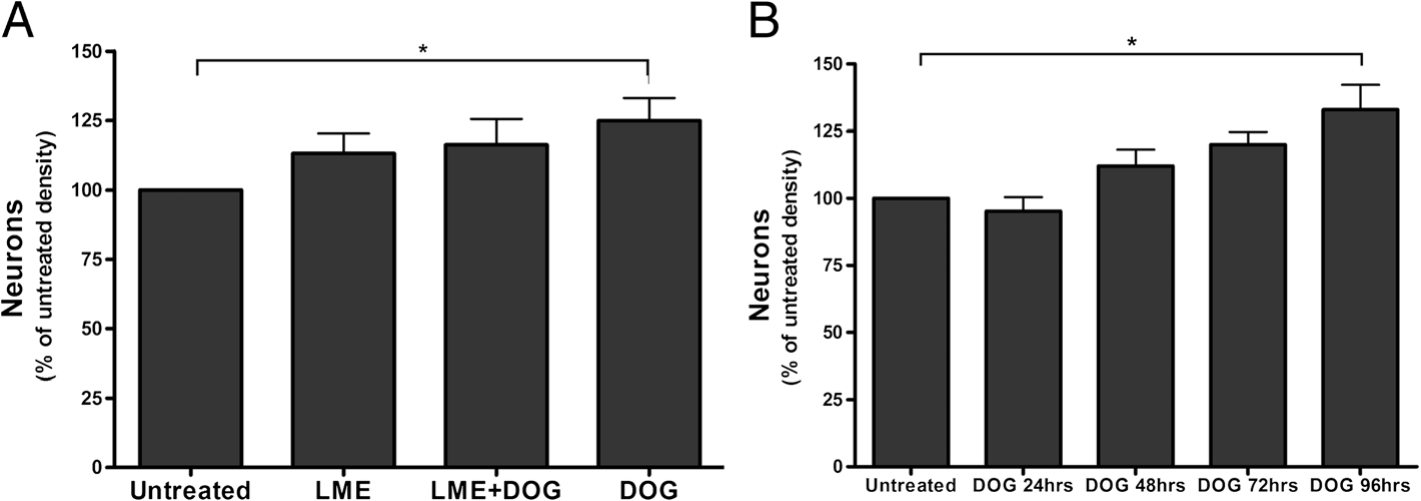
**Effects of deoxyglucose on neuronal density. (A)** Neuronal–glial cultures were treated ± l-leucine-methyl ester (LME) for 4 hours to remove microglia, and then treated ±10 mM deoxyglucose (DOG) for 96 hours, and total (live and dead) neuronal density was counted. Prior depletion of microglia with LME prevented the neuroprotective effect of DOG. **(B)** Neuronal–glial cultures were untreated or treated with 10 mM DOG at 7, 8, 9 or 10 days *in vitro* (DIV), and then total (live and dead) neuronal density measured at 11 DIV. Data presented as mean ± standard error of the mean for ≥ 3 independent experiments. Statistically significant differences between total neuronal densities: *P < 0.05.

To test whether DOG was neurotoxic or neuroprotective, we added 10 mM DOG to primary co-cultures of neurons and glia from rat cerebellum for 96 hours (from 7 to 11 DIV), and observed that there was an apparent increase in the density of live neurons compared with the untreated condition (Figure 1A,D). The density of live microglia substantially decreased over 96 hours of DOG treatment (Figure 1B) but there was no effect on astrocytes (Figure 1C). Thus, in response to DOG, the density of neurons apparently increased by about 30% (relative to untreated cultures) and microglia were almost eliminated, while astrocytes were unaffected. The effect on neuronal density was dependent on the time of incubation with DOG (Figure 2B), with more live neurons surviving with longer treatments with DOG.

The apparent increase in neuronal density induced by DOG could be due to preventing any spontaneous neuronal loss occurring in the untreated cultures, because long-term culture of neurons in the presence of microglia can result in spontaneous neuronal loss [20,21]. To test whether spontaneous neuronal loss was occurring in the untreated cultures, we quantified neuronal density between 7 and 11 DIV, and confirmed that neuronal loss was occurring in the untreated cultures (from 407.5 ± 43.5 to 293.5 ± 4.5). We imaged the same cells at 7 DIV and at 11 DIV, confirming that there is a loss of neurons (Figure 1E).

To determine whether microglia were involved in this neuronal loss over time, we treated the neuronal–glial cultures with 50 mM LME for 4 hours to specifically eliminate microglia [25]. We observed that when microglia were eliminated at 7 DIV, there were more live neurons by 11 DIV, and that addition of DOG from 7 to 11 DIV no longer increased neuronal density in the absence of microglia (Figure 2A). Elimination of microglia is thus sufficient to prevent spontaneous neuronal loss in culture and to prevent the protection of those neurons by DOG. As DOG also depletes microglia from the cultures (Figure 1B), the neuronal protection by DOG appears to be due to DOG depleting the microglia and thereby preventing the spontaneous neuronal loss.

### Deoxyglucose prevents inflammatory neuronal loss induced by amyloid beta or trauma, but exacerbates hypoxic neuronal death

To test whether DOG may be neuroprotective or neurotoxic in particular neurological diseases and conditions, we set up three different pathological models in culture that relate to Alzheimer’s disease, brain trauma and stroke.

**Figure 3:**
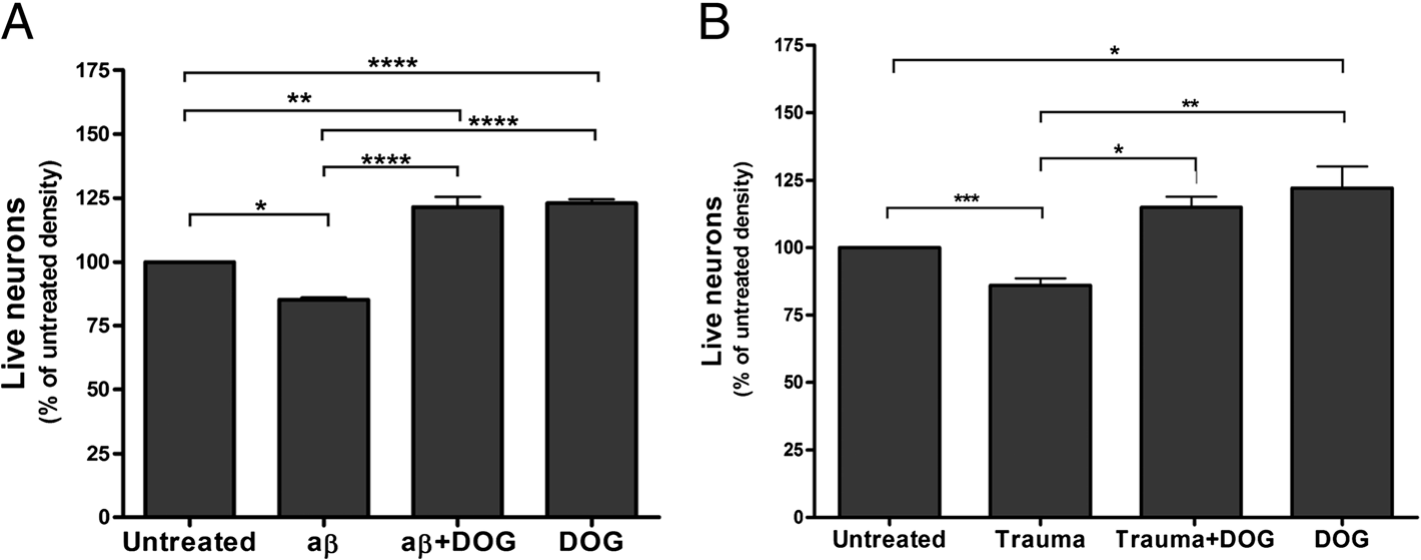
**Deoxyglucose can prevent loss of live neurons induced by amyloid beta and disrupted neurons. (A)** Deoxyglucose (DOG) prevented the neuronal loss induced by amyloid beta (Aβ; 250 nM for 96 hours). **(B)** DOG prevented the neuronal loss induced by adding disrupted neurons (30 μg protein for 96 hours – Trauma). Data presented as mean ± standard error of the mean for ≥ 3 independent experiments. Statistically significant differences between live neuron densities:*P < 0.05, **P < 0.01, ***P < 0.001, ****P < 0.0001.

We have previously reported that nanomolar levels of Aβ induce neuronal loss over 3 days in neuronal–glial co-cultures that is prevented if microglia are eliminated by LME [17,26]. We thus tested whether DOG could prevent this neuronal loss, and indeed found that 10 mM DOG prevented the neuronal loss induced by 250 nM Aβ (Figure 3A). Aβ-induced neuronal loss, when mediated by microglia, can thus be prevented by DOG, presumably by eliminating the microglia.

Brain trauma can cause delayed neuronal damage mediated by microglia [18,19]. We modeled brain trauma *in vitro* by adding disrupted neurons to live neuronal–glial co-cultures, and found that this increased the loss of live neurons over 96 hours (Figure 3B). Disrupted neurons were prepared by scraping and homogenizing neuronal–glial cultures (which are 85% neurons), resulting in cellular debris and released content. Addition of 10 mM DOG prevented the neuronal loss induced by disrupted neurons (Figure 3B). DOG might thus be neuroprotective in brain trauma by depleting microglia.

**Figure 4:**
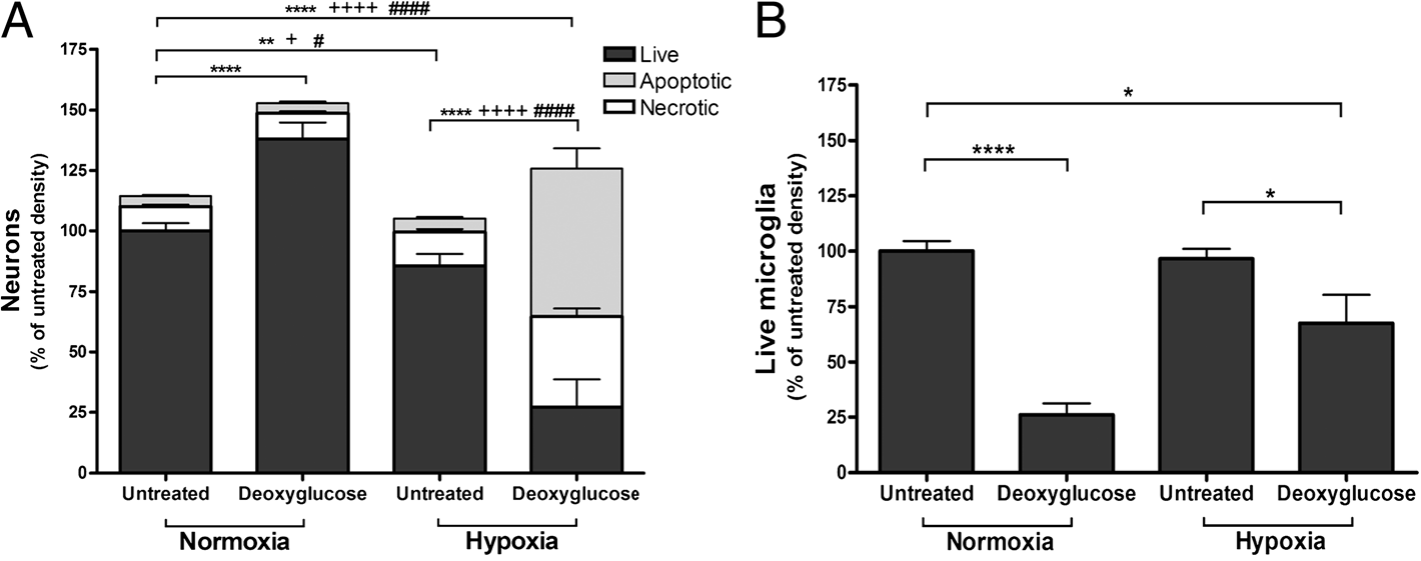
**Deoxyglucose results in neuronal death with hypoxia. (A)** Deoxyglucose increased the neuronal death induced by hypoxia (2% oxygen for 12 hours followed by 96 hours of normoxia; that is, 21% oxygen). **(B)** Density of live microglia in the previous experiment **(A)**. Data presented as mean ± standard error of the mean for ≥ 3 independent experiments. *Live cells, ^#^necrotic cells, ^+^apoptotic cells: *^, #, +^*P* < 0.05, **P < 0.01, ****^, ####, ++++^*P* < 0.0001.

A number of brain pathologies, such as stroke, vascular dementia and trauma, are associated with hypoxia, so we tested whether DOG could prevent or exacerbate hypoxia-induced neuronal death. Moreover, stroke can be ischemic (decrease of blood flow), which can be mimicked *in vitro* by combining hypoxia and glucose deprivation with DOG. Neuronal–glial cultures were exposed to hypoxia (2% oxygen for 12 hours) ± DOG (10 mM) plus 4 days of reoxygenation. As previously, there were significantly more live neurons when DOG was added in normoxic (21% oxygen) conditions (Figure 4A) associated with microglial depletion (Figure 4B). In the absence of DOG, hypoxia induced a small loss of live neurons (Figure 4A). However, in the presence of DOG, hypoxia induced massive neuronal death (Figure 4A), presumably because during hypoxia neurons are more reliant on glycolysis for energy production. DOG can thus be neuroprotective or neurotoxic depending on conditions.

Although DOG decreased microglial levels at both 21% and 2% oxygen, the decrease was less in the latter, hypoxic conditions (Figure 4B). We do not know the reason for this – perhaps oxygen-derived radicals are involved in the cell death, or hypoxia upregulates remaining glycolysis, or the neuronal death occurring in these conditions causes microglial proliferation.

### Microglia are killed by deoxyglucose via ATP depletion and phagocytosis

**Figure 5:**
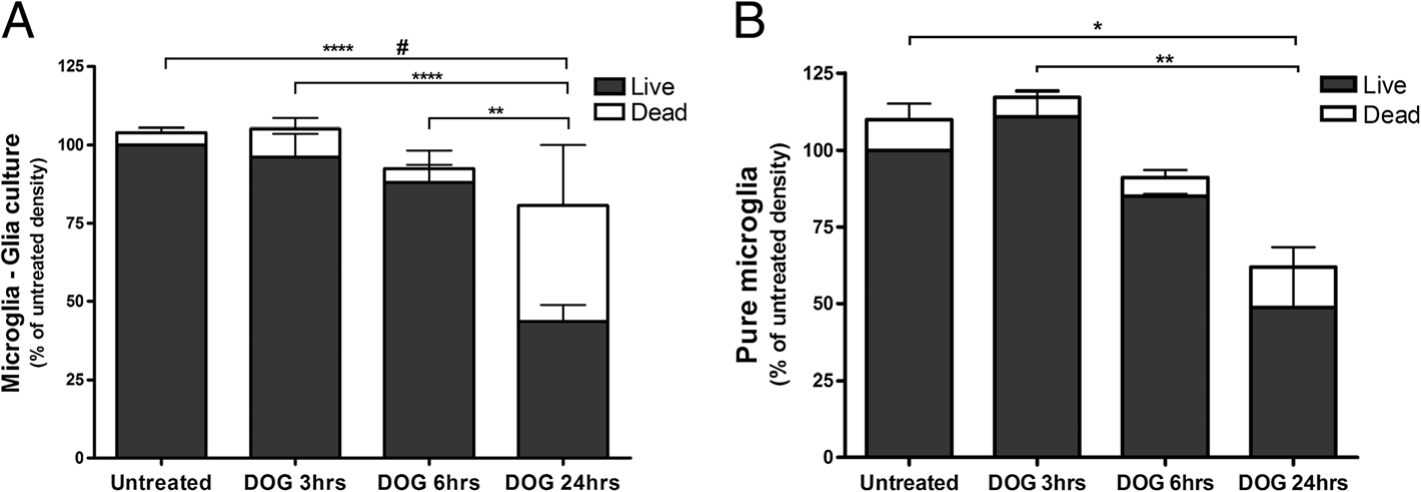
**Microglia are killed by deoxyglucose. (A)** Glial cultures (containing microglia and astrocytes) from rat cortex were treated ±10 mM deoxyglucose (DOG) for the indicated times, and the density of live and dead (necrotic plus apoptotic) microglia were counted. **(B)** Pure primary microglia from rat cortex were treated ±10 mM DOG for the indicated times and the density of live and dead (necrotic plus apoptotic) neurons were counted. Data presented as mean ± standard error of the mean for ≥ 3 independent experiments.*Live cells, ^#^dead cells: *^, #^*P* < 0.05, **P < 0.01, ***P < 0.001, ****P < 0.0001.

**Figure 6:**
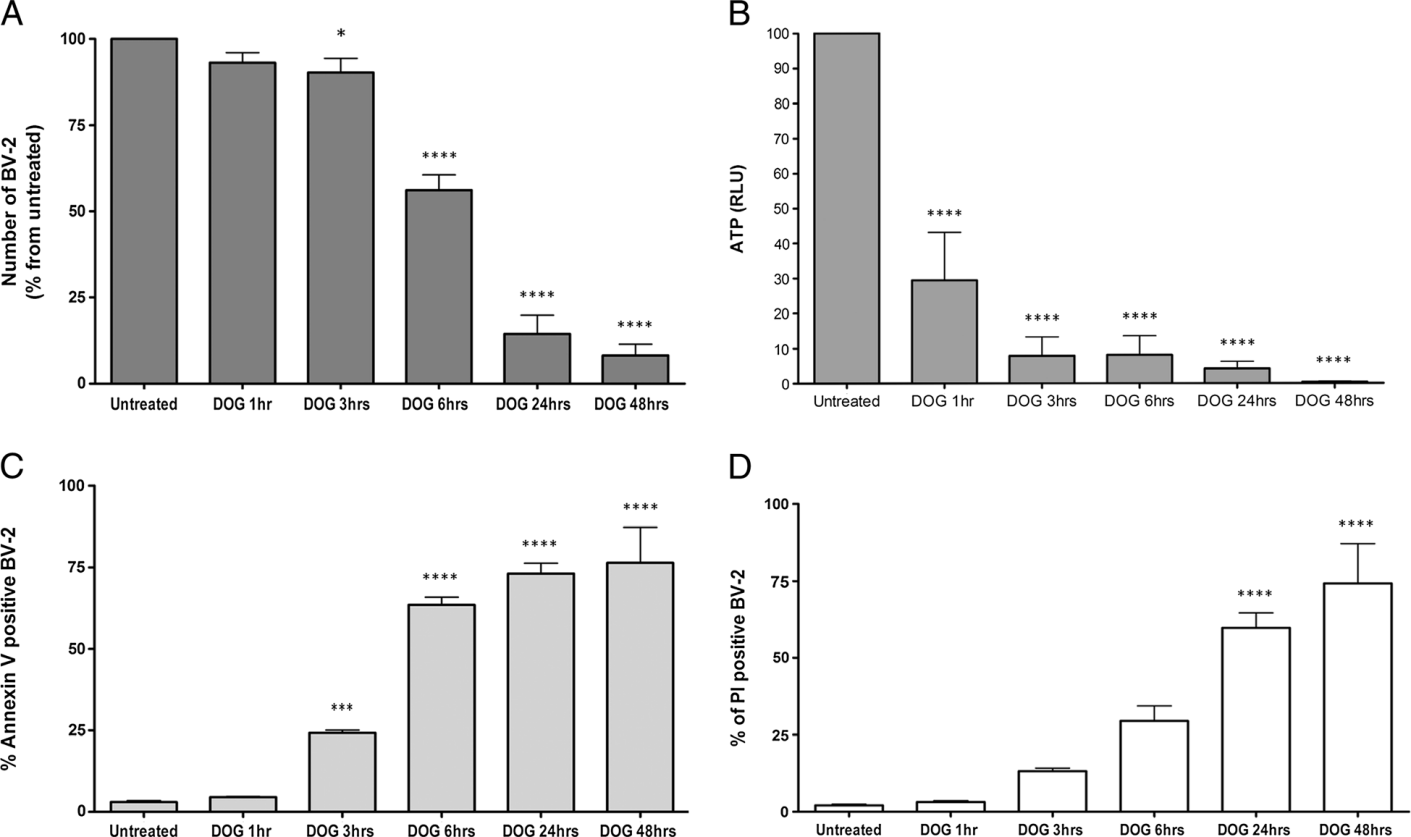
**Deoxyglucose rapidly depletes ATP prior to microglial death. (A)** BV-2 density at different time points (0, 1 hour, 3 hours, 6 hours, 24 hours, 48 hours) after addition of 10 mM deoxyglucose (DOG) measured by flow cytometry. **(B)** Cellular ATP levels determined with luciferin/luciferase at the same time points. RLU, relative light units. **(C)** Percentage of Annexin V-positive BV-2 cells measured by flow cytometry at the same time points. **(D)** Percentage of propidium iodide (PI)-positive BV-2 cells measured by flow cytometry at the same time points. Data presented as mean ± standard error of the mean for ≥ 3 independent experiments.*P < 0.05, ***P < 0.001, ****P < 0.0001.

DOG depleted microglia in neuronal–glial cultures over 96 hours (Figure 1B), so we tested whether DOG would kill or deplete microglia in a glial culture (Figure 5A), a pure microglial culture (Figure 5B) or the microglial cell line BV-2 (Figure 6). Twenty-four hours of DOG treatment caused a significant decrease in microglial density in all three cultures and significant increases in microglial necrosis (measured by propidium iodide uptake) in glial cultures and the BV-2 cell line (Figures 5 and 6). The decrease in microglial density in BV-2 cultures was substantially greater with 10 mM DOG than with 1 or 5 mM DOG (Additional file 1: Figure S1), so we used 10 mM DOG in subsequent experiments. The proportion of microglia that were chromatin condensed but had not taken up propidium iodide (a measure of apoptosis) was low in all conditions. The proportion of DOG-treated BV-2 microglia that exposed phosphatidylserine (measured by Annexin V binding; Figure 6C) increased with time, preceding the cells becoming necrotic (measured by propidium iodide uptake; Figure 6D), which could be due to apoptosis or energy depletion-induced phosphatidylserine exposure.

We tested whether the broad-spectrum caspase inhibitors ZVal-Ala-d,l-Asp(OMe)-fluoromethylketone and bocaspartyl (OMe)-fluoromethylketone could prevent DOG-induced death of microglia. However, there was no such protection (Additional file 2: Table S1), suggesting that apoptosis is not involved.

DOG can kill cancer cells by interfering with N-linked glycosylation, which is reversible by exogenous addition of mannose [27,28]. We tested whether addition of 2 mM mannose for 48 hours could prevent DOG-induced microglial loss in primary glial cultures, but found no effect of mannose on microglia loss (Additional file 2: Table S1).

DOG can activate sirtuins, such as Sirtuin 1, in cells via inhibiting glycolysis and increasing NAD^+^ levels. Sirtuin 1 is a NAD^+^-dependent histone deacetylase that is inhibited by nicotinamide. However, we found that nicotinamide (at 100, 300 or 600 μM) was not able to prevent DOG-induced microglial death (Additional file 2: Table S1), suggesting that sirtuins are not involved in this death.

Autophagy can be activated by DOG in some cells [29]. We tested whether the autophagy inhibitor chloroquine (10 or 25 μM) could prevent DOG-induced microglia death, but found no effect (Additional file 2: Table S1).

DOG can deplete cellular ATP by inhibition of glycolysis [1,2]. We therefore tested whether DOG could deplete ATP in microglia prior to any cell death or loss. Indeed, we found that DOG caused marked ATP depletion after just 1 hour of DOG treatment of pure, primary microglia (Additional file 3: Figure S2A) or BV-2 microglia (Figure 6B) prior to any increase in cell death (Figure 6D). This suggests that DOG induces death of microglia by ATP depletion, and that microglia are dependent on glycolysis for energy generation.

To test whether DOG could stimulate or inhibit the activation of microglia prior to any significant microglial death, we measured TNFα levels in the culture medium of pure, primary microglia at 3 hours after addition of 10 mM DOG. We observed a small increase in TNFα production/release (Additional file 3: Figure S2B) prior to any increase in cell death (Figure 5B).

**Figure 7:**
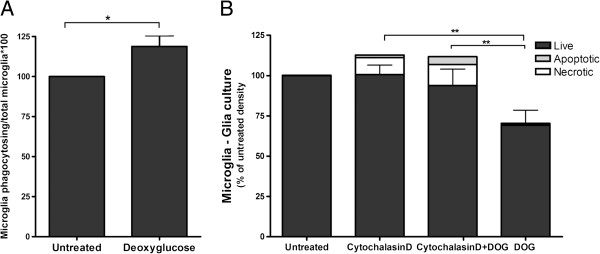
**Microglial phagocytosis contributes to the loss of microglia induced by deoxyglucose. (A)** Pure primary microglia were treated ± deoxyglucose (DOG; 10 mM) for 21 hours, and were then incubated with 5 μm carboxylate-modified latex microspheres for 2 hours, and the number of beads phagocytosed per cell quantified. **(B)** The phagocytosis inhibitor cytochalasin D (1 μM) decreased the loss of microglia measured 6 hours after the addition of DOG (10 mM) in glial cultures. Data presented as mean ± standard error of the mean for ≥ 3 independent experiments. *Total microglia: *P < 0.05, **P < 0.01.

As the loss of microglia could be due to phagocytosis by other microglia, we tested whether deoxyglucose could stimulate or inhibit microglial phagocytosis. Pure microglia were treated with 10 mM deoxyglucose for 21 hours and then 5 μm carboxylate-modified latex microspheres were added for 2 hours. We observed a very small increase in phagocytosis of microspheres with DOG treatment (Figure 7A).

Dead and dying cells can be rapidly removed by phagocytosis, and microglia are particularly active in this type of phagocytosis [15]. To test whether phagocytosis was involved in the microglial loss induced by DOG, we added an inhibitor of phagocytosis: cytochalasin D (1 μM) together with 10 mM DOG for 6 hours. Cytochalasin D delayed the loss of microglia induced by DOG treatment (Figure 7B), indicating that phagocytosis may be involved in the microglial loss. Annexin V is a phosphatidylserine-binding protein, which when added extracellularly can block phosphatidylserine-mediated phagocytosis. However, addition of Annexin V (at concentrations that we have previously found to block microglial phagocytosis: 100 nM) could not prevent the microglial loss induced by DOG (Additional file 2: Table S1), suggesting that eat-me signals other than phosphatidylserine are involved in the phagocytosis of dying microglia by microglia.

## Discussion

Addition of DOG depleted microglia, protected neurons and had no effect on astrocyte viability. The mechanism by which DOG depleted microglia appears to be mainly via directly killing the microglia, probably via inhibition of glycolysis and ATP depletion, inducing microglial necrosis and their phagocytosis by other microglia. Although DOG did increase the number of necrotic microglia, this increase was less than the decrease in total microglial numbers, suggesting that necrotic or prenecrotic cells are removed rapidly by viable microglia. Consistent with this, we found that DOG did not inhibit microglial phagocytosis, and that inhibition of phagocytosis by cytochalasin D delayed the DOG-induced microglial loss. It is not clear why inhibition of glycolysis by DOG specifically kills microglia and not neurons or astrocytes, although a simple explanation would be that neurons derive most of their ATP from mitochondria [30], while microglia/macrophages derive most of their ATP from glycolysis, particularly when activated [31]. There are no previous reports that DOG kills microglia.

The mechanism by which DOG protected neurons appears to be via depletion of microglia based on our findings that DOG did deplete microglia, that depleting microglia with LME also protected neurons, and that depleting microglia with LME prevented DOG protecting neurons. In the longer term, any DOG inhibition of microglial proliferation might also contribute to microglial depletion. DOG might in principle inhibit microglial activation – but we found that DOG mildly increased TNFα levels and phagocytosis, making this potential explanation for the neuroprotection unlikely. DOG does not directly inhibit the pentose phosphate pathway, but can indirectly perturb it [32] or increase it by inhibiting glycolysis, which might contribute to cell death, although this pathway is normally thought to be protective. Recent evidence suggests that modulating mitochondria can decrease the proinflammatory status of microglia [33,34]. Park and colleagues found that inhibition of mitochondrial fission and reactive oxygen species generation attenuated the production of proinflammatory mediators [33]. On the other hand, Voloboueva and colleagues observed that overexpressing a mitochondrial chaperone glucose-regulated protein 75 decreased microglial activation [34].

DOG has shown some promise in treating animal models of epilepsy [3,4], Alzheimer’s disease [8] and Parkinson’s disease [7], and while other mechanisms are not excluded, it is possible that DOG-induced depletion of microglia may contribute to this protection *in vivo*. Obviously it would be useful to know whether DOG therapy *in vivo* does in fact deplete brain microglia, but there is currently no information on this. DOG has been used to mimic the metabolic effects of dietary restriction in reducing aging (for example [7,11]), so it would be interesting to know whether dietary restriction also depletes microglia in the brain because dietary restriction has been shown to improve cognitive function in aged rodents and humans [35].

DOG prevented Aβ-induced neuronal loss, which we have previously shown to be dependent on microglia [17,26], so this is consistent with the DOG protection being due to depletion of microglia. DOG prevented the neuronal loss induced by disrupted neurons, presumably by microglial depletion. DOG also protected against spontaneous neuronal loss in these neuronal–glial cultures. We do not know what causes this spontaneous neuronal loss, but in general it is more likely to occur with longer-term culture (more DIV) and when there are more dead neurons in the culture. As addition of disrupted/dead neurons to the culture can induce neuronal loss, it may be that this spontaneous neuronal loss is driven by dead neurons present in the culture. Whatever the cause, our finding that DOG can prevent this spontaneous neuronal loss may be of practical value in culturing neurons in the longer term.

Our finding that DOG does not protect against hypoxia-induced neuronal loss, but rather greatly potentiates hypoxia-induced neuronal death, is not surprising given that inhibition of both glycolysis and mitochondrial respiration will cause ATP depletion in all cells. However, this does highlight the dangers of using DOG as a brain therapy given that hypoxia occurs in a variety of brain pathologies such as stroke, vascular dementia and trauma. On the other hand, because brain hypoxia can be monitored non-invasively in patients, this danger could in principle be minimized. Also DOG can be protective against ischemic damage if the brain is hyperglycemic by preventing excessive lactate production in such conditions [36].

## Conclusions

We conclude that DOG causes microglial death by ATP depletion, resulting in microglial loss by phagocytosis, and this can protect neurons from inflammatory neurodegeneration. DOG may thus be beneficial in brain pathologies mediated by microglia, but not where hypoxia is involved.

## Abbreviations

Aβ: amyloid beta; DIV: days *in vitro*; DOG: 2-deoxy-d-glucose; LME: L-leucine-methyl ester; TNFα: tumor necrosis factor alpha.

## Competing interests

The authors declare that they have no competing interests.

## Authors’ contributions

AV carried out all the experiments, and contributed to the design and analysis of the experiments, and contributed to writing the manuscript. GCB contributed to the design and analysis of the experiments, and contributed to writing the manuscript. Both authors read and approved the final manuscript.

## Supplementary Material

Additional file 1: Figure S1 showing the effect of different doses (1 mM, 5 mM, 10 mM) of DOG on BV-2 density measured by flow cytometry. ***P < 0.001. Click here for file

Additional file 2: Table S1 presenting a list of the compounds tested that did not prevent death of microglia induced by DOG. zVAD, ZVal-Ala-D,L-Asp(OMe)-fluoromethylketone; BAF, bocaspartyl (OMe)-fluoromethylketone; PS, phosphatidylserine. Click here for file

Additional file 3: Figure S2 showing DOG rapidly depletes ATP and inflammatory activates microglia. (A) ATP levels were determined in pure primary microglia cultured from rat cortex and treated with DOG (10 mM) for 1 hour. RLU, relative light units. (B) TNFα levels were measured in the culture medium of pure primary microglia treated ± 10 mM deoxyglucose for 3 hours. Data presented as mean ± standard error of the mean for ≥ 3 independent experiments.*P < 0.05, **P < 0.01. Click here for file
