# Supplementary figures and images for: Deoxyglucose prevents neurodegeneration in culture by eliminating microglia
Source: J Neuroinflammation. 2014 Mar 26;11:58. doi: 10.1186/1742-2094-11-58 (PMC3986974; doi:10.1186/1742-2094-11-58)

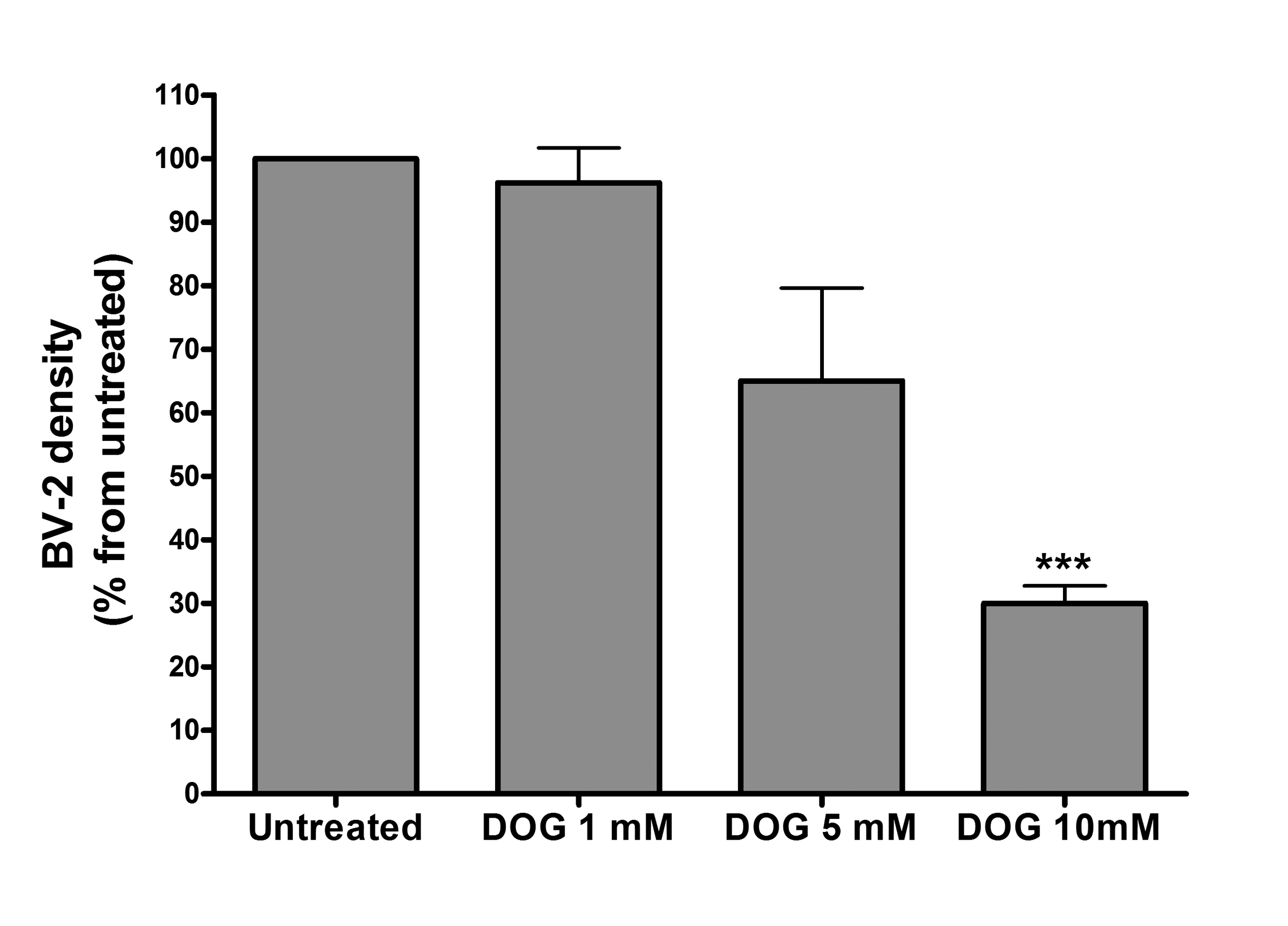

Supplement: Additional file 1: Figure S1 — showing the effect of different doses (1 mM, 5 mM, 10 mM) of DOG on BV-2 density measured by flow cytometry. ***P < 0.001. [file 1742-2094-11-58-S1.tiff]

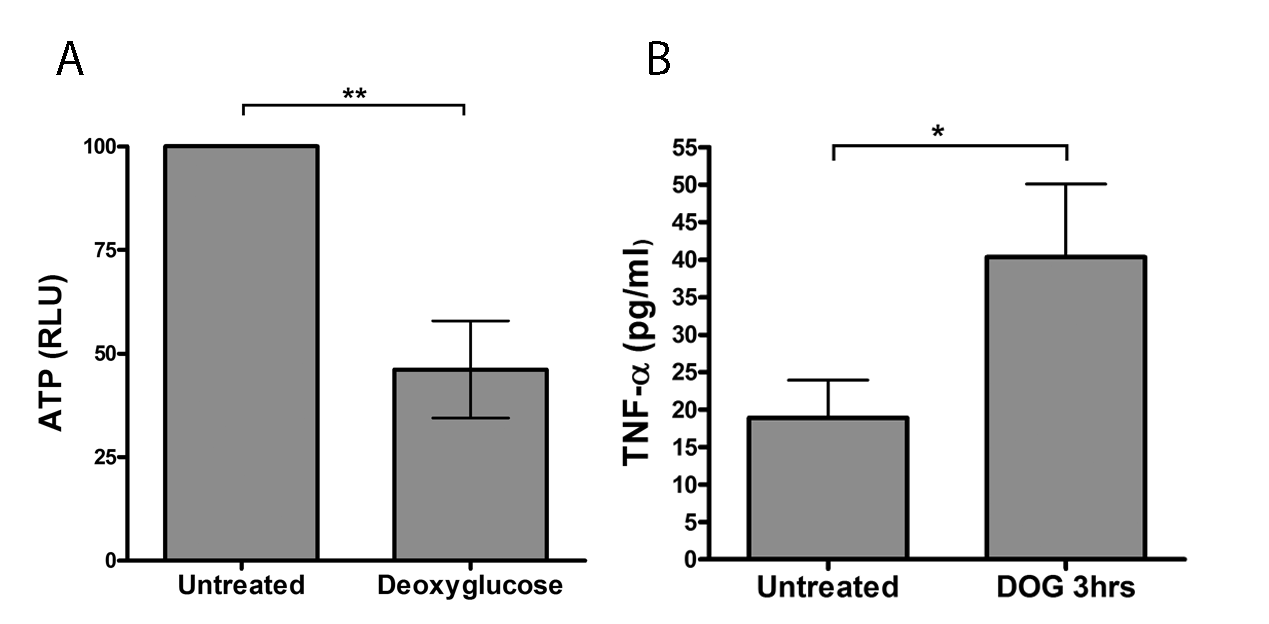

Supplement: Additional file 3: Figure S2 — showing DOG rapidly depletes ATP and inflammatory activates microglia. (A) ATP levels were determined in pure primary microglia cultured from rat cortex and treated with DOG (10 mM) for 1 hour. RLU, relative light units. (B) TNFα levels were measured in the culture medium of pure primary microglia treated ± 10 mM deoxyglucose for 3 hours. Data presented as mean ± standard error of the mean for ≥ 3 independent experiments.*P < 0.05, **P < 0.01. [file 1742-2094-11-58-S3.tiff]
