# Supplementary material for: Deoxyglucose prevents neurodegeneration in culture by eliminating microglia
Source: J Neuroinflammation. 2014 Mar 26;11:58. doi: 10.1186/1742-2094-11-58 (PMC3986974; doi:10.1186/1742-2094-11-58)
Supplement: Additional file 2: Table S1 — presenting a list of the compounds tested that did not prevent death of microglia induced by DOG. zVAD, ZVal-Ala-D,L-Asp(OMe)-fluoromethylketone; BAF, bocaspartyl (OMe)-fluoromethylketone; PS, phosphatidylserine. [file 1742-2094-11-58-S2.doc]

**Additional file 3**

| **Compound** | **Objective** | **Doses** | **Type of cells** | **Length of treatment** | **Microglia density**  **(mean ±SEM)** | **Result** |
| --- | --- | --- | --- | --- | --- | --- |
| **Mannose** | To test whether DOG kills cells by competing with mannose for glycosylation. | 2mM | Pure primary microglia | 48 hours | **Untreated:** 66±7.6  **DOG:** 3.5±0.9  **Mannose:** 63.6±6  **DOG+Mannose:** 4.2±1 | No effect |
| **BAF & zVAD** | Caspase inhibitors. To test whether DOG-mediated microglia death is apoptotic. | 100 µM BAF;  50 µM zVAD | Microglia in neuron-glia co-cultures | 16 hours | **Untreated:** 12.5±1  **DOG:** 5±1.2  **DOG+BAF:** 5.5±1.5  **DOG+zVAD:** 7±2.6 | No effect |
| **Chloroquine** | Autophagy inhibitor. To test whether DOG-mediated microglia death is by autophagy | 10 µM  25 µM | Microglia in glial cultures | 96 hours | **Untreated:** 48.4± 3.3  **DOG:** 26.1±5.5  **DOG+Chloroquine 10µM:** 29±4.1  **DOG+Chloroquine 25μM:** 28.4±2.5 | No effect |
| **Nicotinamide**  **Annexin V** | To test whether inhibiting sirtuins prevents microglial death  To test whether blocking PS exposure prevents microglial death | 100 µM  100nM | Microglia in glial cultures  Microglia in glial cultures | 96 hours  6 hours | **Untreated:** 22.2±2.7  **DOG:** 0.9±0.5  **Nicotinamide:** 24.5±2  **DOG+nicotinamide:** 0.9±0.3  **Untreated:** 77.1±23.9  **DOG:** 47.7±31.2  **Annexin V:** 99.1±54.9  **DOG+AnnexinV:** 67.5±33 | No effect  No effect |

**Table S1.** List of the compounds tested that did not prevent death of microglia induced by deoxyglucose (DOG). zVAD (ZVal-Ala-D,L-Asp(OMe)-fluoromethylketone); Bocaspartyl (OMe)-fluoromethyketone (BAF); PS (phosphatidyl serine).
